# Perils of Precisely Equal Group Size in Randomised Controlled Trials

**DOI:** 10.3390/nursrep15120422

**Published:** 2025-11-28

**Authors:** Richard Gray, Daniel Bressington, Bridgina Mackay, Martin Jones, David R. Thompson

**Affiliations:** 1 School of Nursing and Midwifery, La Trobe University, Melbourne, VIC 3004, Australia; bridgina.mackay@acu.edu.au; 2 Faculty of Nursing, Chiang Mai University, Chiang Mai 50200, Thailand; daniel.bressington@cmu.ac.th; 3 School of Nursing and Midwifery, Edith Cowan University, Perth, WA 6050, Australia; martin.jones@ecu.edu.au; 4 School of Nursing and Midwifery, Queens University Belfast, Belfast BT9 7BL, UK; david.thompson@qub.ac.uk

The randomised controlled trial is the best approach for testing the effectiveness of a new intervention [1]. Randomisation is core to this study design and is intended to ensure that participant characteristics (both observed and unobserved) are essentially the same in each arm of the trial. If a new intervention is given to one group and not the other, and there is an observed difference in outcome, effectiveness can be inferred.

Simple randomisation requires that participants have an equal chance of being allocated to each arm of the trial. In a two-arm trial there should be a 50:50 chance—equivalent to flipping a coin—of being allocated to the experimental or control group.

We encourage readers to browse through randomised controlled trials published in nursing journals and look at the CONSORT flow diagram [2] (a visual representation of participant flow through a study). You may notice a pattern: in many nursing trials, the number of participants allocated to each group is identical [3]. You may well be thinking, ‘don’t the two groups need to have the same number of participants?’, to which the answer is no; in fact, esteemed trialists have described this idea as ‘cosmetic’, not methodological [4]. In this Editorial, we want to consider the perilous pursuit of trying to achieve precisely equal group sizes in randomised trials. We should note at this point that we are aware of restrictive (block) approaches to randomisation which deliberately intend to balance group numbers. However, these approaches—commonly used in medical studies [5]—are rarely used in nursing trials (only around 22% of nursing trials use block randomisation [6]). There are also issues with predictability in block randomisation, which can result in trial investigators being able to anticipate which group a newly recruited participant will be allocated, but this is possibly a topic for another time [6].

Several years ago, we reviewed 148 trials published in nursing science journals in 2017 (with a different aim). Strikingly, the authors of 100 (68%) trials (most using simple randomisation) had precisely equal group sizes, which we thought, at the time, was rather intriguing [3]. A few years on, the issue seemingly persists. To illustrate (not prove) the point, we browsed SCOPUS to find a recently published randomised study reported in a nursing journal. The first we came across was by Turan and colleagues [7] who report a clinical trial involving 66 caregivers of palliative care patients and published in *Nursing Open*, with the authors concluding that progressive muscle relaxation exercises reduced stress and anxiety. According to the CONSORT flow diagram, there were 33 participants in each of the two trial arms—exactly equal group sizes. According to the manuscript, the authors used a computerised (simple) randomisation process (coin toss). What are the chances of flipping a coin 66 times and obtaining 33 heads and 33 tails? Give it a try! We did some (not complex) maths: the probability of obtaining this result is about 1 in 10. Not impossible, but unlikely. Curiously, the authors do not mention equal group size as an issue in the manuscript suggesting that neither they, the reviewers, nor journal editors thought it was a problem—but it is.

The questions you may – or perhaps should – be asking yourself are ‘how’ and/or ‘so what’ if a trial has equal groups? The answer to ‘how’ is by either by allocating and not randomising participants to trial arms or by having a restricted allocation sequence. The ‘so what’ is because researchers either know or can work out (decipher) group allocation.

Consider a hypothetical trial where participants are randomised by flipping a coin to allocate them to a particular group. As the trial progresses, they realise that they have recruited the necessary number of participants in the control group but still need to recruit a further X people to complete the trial. Should they continue to flip a coin, likely resulting in an unbalanced group? The simple answer is yes; but often, to balance groups, researchers *allocate* the remaining participants to the other (in this example, the experimental) group. Because they know which group participants will be in, they will (either consciously or subconsciously) tend to recruit people they think will respond to the new intervention, thus, introducing bias.

In the Turan et al. trial [7] the researchers needed to recruit 66 participants. They used a computer software package to generate an allocation sequence with 33 participants in each group. Once 66 participants have been recruited they stop the trial, achieving two equal groups. The problem is that researchers know the number of participants that will be in each group. Consequently, as the trial progresses, it will become easier for them to make an educated guess as to which group the next participant will be allocated to. Think about it for a moment. If, after 40 participants have been recruited, 25 are allocated to the control group (remember that allocation sequences are randomly generated, so this can happen with simple randomisation), it is a fair guess that the next individual recruited will be allocated to the experimental group. Researchers may therefore select participants whom they think are more likely to respond to the experimental intervention, again introducing bias.

It is a plausible hypothesis that manipulation introduced to generate precisely equal group sizes introduces important bias into trial design, plausibly resulting in a type 1 error (false positive results). This hypothesis (are trials with precisely equal group sizes more likely to report positive results?) certainly warrants further investigation (and we are on to this).

We randomise in trials to reduce the risk of selection bias and ensure the comparability of comparison groups [8]. The misplaced need to produce precisely equal groups threatens this bedrock principle of the randomised controlled trial. Do we have a problem in nursing as an ‘emerging’ discipline conducting randomised controlled trials? The misplaced need to achieve balanced groups, we speculate, is motivated by the ‘publish or perish’ culture that nursing has seemingly bought wholesale into; trials need to ‘look pretty’ to be published in the ‘best’ journal. The training of nurse researchers in how to conduct clinical trials is probably inadequate, and we strongly suspect that this may explain the high prevalence of poor randomisation procedures we observe in the manuscripts we have read (reviewed and edited). It would, perhaps, be helpful to talk to nurse researchers conducting trials to obtain an understanding of how well they understand randomisation procedures and what their training needs might be.

It is frustrating that these practices still occur, when mechanisms for randomising in clinical trials have been well established for decades. Many universities and health services have clinical trial units that will support researchers in randomising participants; there are also a range of inexpensive web-based randomisation services (e.g., https://www.sealedenvelope.com/ accessed 28 November 2025) that can be used. The ‘DIY’ approach to randomisation is sloppy. Just stop!

Just as a fun aside, there is a risk with simple randomisation in small trials that all (or the preponderance of) the participants are allocated to just one group. We do wonder, as a more serious point, if this might overly concern researchers leading them to design trial mechanisms that achieve equal group sizes. The risk is small; for example, in a trial involving 20 participants, there is about a 1 in 500,000 chance that all participants will be allocated to the same group, which we think you will agree is unlikely. So do not stress about this risk.

Randomisation procedures in many of the clinical trials we are undertaking and reporting as nurses is not the ‘gold standard’. We must do better. By way of this Editorial, we are asking researchers, institutions, journal editors, and reviewers to be much more proactive in improving randomisation processes. As a group of journal editors, we commit to ensuring trial authors fully explain how they randomised and clearly acknowledge any resulting bias. Ultimately, this is a patient safety issue; interventions should not be implemented into practice if the evidence showing they are safe and effective is flawed.
